# Expectations Regarding Aging After Relocation to an Assisted Living Community

**DOI:** 10.1093/geroni/igaa057.1233

**Published:** 2020-12-16

**Authors:** Evan Plys

**Affiliations:** University of Colorado Denver - Anschutz Medical Campus, Denver, Colorado, United States

## Abstract

This study adds to the growing literature on attitudes toward aging in older adulthood by using a multidimensional measure to assess heterogeneous profiles of expectations regarding aging (ERA) in a sample of assisted living (AL) residents. The author analyzed secondary data from a cross-sectional quantitative study consisting of 202 residents of 21 ALs. Participants were mostly female (72%), white (90%), and widowed (59%); ages ranged from 51 to 100 (M = 83.05, SD = 10.32). Hierarchical Cluster Analysis identified four subgroups: (1) “healthy agers”, n = 54, 27%, characterized by high physical, emotional, and cognitive ERA; (2) “cognitively intact”, n = 41, 20%, characterized by low physical, low emotional, and high cognitive ERA; (3) “coping with decline”, n = 56, 28%, characterized by moderate physical, high emotional, and low cognitive ERA; and (4) “unhealthy agers”, n = 51, 25%, characterized by low physical, emotional, and cognitive ERA. Subgroups varied by mental health (healthy agers > unhealthy agers), cognitive ability (cognitively intact > coping with decline), and activity participation (coping with decline > unhealthy agers). Surprisingly, groups did not differ based on social support from co-residents, staff, or family. Results demonstrate that distinct subgroups of ERA exist among AL residents, supporting the utility of assessing ERA as a multidimensional construct in this setting. In addition, findings suggest that expecting to retain health and ability in at least one domain may protect against behavioral consequences of negative ERA. The author also discusses implications for future research and clinical practice in AL.

